# Burden and correlates of non-communicable-diseases among rural residents: a cross-sectional study in Hebei, China

**DOI:** 10.1186/s12889-015-1916-x

**Published:** 2015-06-20

**Authors:** Junjun Yang, Wenya Yu, Qiang Zhou, Tanmay Mahapatra, Yiqiu Li, Xiaoyan Zhang, Lei Chen, Sanchita Mahapatra, Yuying Yan, Weiming Tang

**Affiliations:** Department of Laboratory, Wuxi No. 2 People’s Hospital Affiliated to Nanjing Medical University, Wuxi, 214002 China; Shijiazhuang Center for Disease Control and Prevention, 050011 Shijiazhuang, Hebei China; The Bethune Medical NCO College, Shijiazhuang, Hebei 050081 China; Department of Epidemiology, Fielding School of Public Health, University of California, Los Angeles, 90066 Los Angeles, USA; Department of STI Control, Guangdong Provincial Center for Skin Diseases and STI Control, Guangzhou, 510095 China; University of North Carolina, Project-China, No. 2 Lujing Road, Guangzhou, 510095 China

**Keywords:** Non-communicable diseases, Demographic correlates, NCDs, Chronic diseases, Unhealthy behaviors

## Abstract

**Background:**

Burden of non-communicable diseases (NCDs) is increasing rapidly in most of the developing countries including China, even in rural areas. Dearth of representative data called for an investigation to estimate the burden and identify the correlates of NCDs in rural China.

**Methods:**

A cross-sectional study was conducted involving a representative sample of 6003 consenting randomly selected rural residents aged 15 years or more, from 36 villages of Shijiazhuang in Hebei province of China between July 2010 and June 2011. Information on demographics and behavior were collected, body mass index (BMI) and blood pressure were measured and blood samples were tested to diagnose diabetes and hyperlipidemia.

**Results:**

Majority participants were aged < 30 year, married and educated up to junior/senior high school level. Mean age for the 6003 participants was 37.4 ± 14.8. About 55.7 % had BMI of 18.6-24.9. In past 12 months: 19.8 % smoked daily, 41.6 % were exposed to passive smoking, 28.5 % drank alcohol, 10.4 % skipped breakfasts frequently, 82.8 % did never exercise and 25.3 % had psychological disturbances. 51.1 % were hypertensive, 6.7 % were diabetic and 9.2 % had hyperlipidemia. Based on self-reports, cardiovascular diseases (4.5 %), cerebrovascular diseases (2.3 %), cancers (0.2 %), chronic obstructive pulmonary diseases (2 %), orthopedic problems (12.1 %) and gastrointestinal NCDs (7.8 %) were identified among the participants, while proportion of subjects with one, two and three or more NCDs were 43 %, 14.4 % and 5.5 % respectively. Higher odds of having more NCDs were associated with higher BMI (Kg/M^2^), family history of NCDs, daily and past history of smoking and drinking, passive smoking, lack of exercise, skipping breakfast and psychological disturbances.

**Conclusion:**

Despite limitations associated with cross-sectional design and self-reporting, observation in this large sample of rural residents could develop important insights regarding high burden of NCDs in this population. Based on the identified correlates, targeted intervention strategies seem to be required urgently to control NCDs in rural China.

## Background

The burden of non-communicable diseases (NCDs) is rising rapidly and remains the leading cause of mortality in the world [1]. As per WHO estimates, more than 36 million people die each year from NCDs and nearly 80 % of such deaths occur in low and middle income countries [2]. Keeping in mind the current trend, it is predicted that by the year 2020, 73 % of deaths and 60 % of disease burden will be attributable to NCDs globally [3].

With the changing trend of epidemiology of NCDs, the burden of hypertension, diabetes, cancer and other cardiovascular diseases which were once known to be the ailments of affluence are now progressively increasing in developing world much faster than their developed counterparts [4].

In China, the most populous country in the world, the burden of NCDs has reached an epidemic proportion, accounting for an estimated 80 % of total annual deaths and 70 % of its total disease burden [5]. With the progress in rapid economic development, China has confronted an epidemiological transition, as the predominant cause of mortality and morbidity has shifted from infectious diseases to NCDs in China [6]. A recent study in Hubei province of China revealed that NCDs accounted for 86.2 % of total deaths among people aged over 15 years during 2008-2010. Major causes of mortality identified were cerebrovascular diseases, ischaemic heart diseases and neoplasms and compared to urban areas, overall, the standardized mortality rates were found to be higher in rural areas [7]. In another population based survey in Beijing, the prevalence of hypertension was found to be significantly higher among rural populations in 2006 compared to urban population [8]. Available evidences in contemporary scientific literatures indicated that a dramatic increase in the prevalence of potential behavioral risk factors like dietary changes, reduced physical activity, alcohol and tobacco use in recent decades might be the major driving force behind this emergence of NCDs in rural China [6]. Striking differences in social and economic environment between urban and rural areas in this country could also have culminated into a negative impact on the health of older adults in rural areas [9, 10].

Despite conducting several geographically scattered investigations to estimate the occurrence of individual chronic diseases, in lieu of the growing concern about the emergence of NCD epidemic in rural China, investigation involving a representative rural population seemed to be the need of the hour.

In order to estimate the burden of NCDs among rural residents, and to identify their demographic and behavioral correlates, a cross-sectional study was conducted between July 2010 and June 2011, in a rural population of Shijiazhuang in Hebei province of China.

## Methods

### Recruitment

In order to recruit a sample with better representativeness, at the design phase, we used stratified sampling (stratified on county and villages) with probability proportional to size (PPS) sampling method to select about one person per1000 residents of rural areas (to maintain the proportional distribution of the population in the sample) of Shijiazhuang. According to the 2010 population census of China, there were 7,355,238 persons living in the rural areas of Shijiazhuang, and about 5.5 million of them were aged 15 years or more [11]. Thus about 5,500 persons were required to be recruited.

There are 18 counties in Shijiazhuang, among them, four (about 20 %) were selected randomly. Further, 9 villages (about 10 %) were randomly selected from each selected county and thus altogether 36 sampling villages were identified. From the selected villages, required number (proportional to the population of the corresponding villages, about 50 %) of legal residents, who were aged 15 years or more were selected randomly and invited to participate in the study so that after accounting for 10 % assumed non-response 6150 subjects get the invitation. Among these rural residents, those who agreed to participate and provided voluntary informed consent were recruited for the study. The selected participants were invited to go to the community/village centers at each village to attend the study. To reduce the non-response rate, we also visited the people who did not come to the study sites.

### Structured interview

A face-to-face interview using an interviewer-administered structured questionnaire was conducted for each participant to collect information on demographics, anthropometric measurements, related recent behaviors and disease history.

The demographic information included age (continuous and further categorized into Less than 30 years/30–39years/40–49 years/50 years or more), gender (female/male), education level (elementary school or less/junior or senior high school/college and higher) and marital status (never married/ divorced or widowed/ married). Anthropometric measurements included height (in meter), weight (in kilogram,) and body mass index (BMI, weight/height^2^ in kg/m^2^). BMI was further categorized into three groups: 18.5 or less, 18.6 to 24.9, and 25.0 or above.

Recent behaviors were assessed by collecting information about last 12 months on active smoking status (daily smoker/smoker, but not daily/ex-smoker/never smoker), passive smoking status (experienced passive smoking more than 15 min each week, yes/no), exercise (more than 15 min each time, often/rare/never), significant weight change (increased more than 2.5kgs/no change/decreased more than 2.5kgs), labor work in the farm (never/ yes, but no busy season/ yes and have busy season).

The mental state of the participants was assessed by the following four questions: whether 1) feeling alone; 2) feeling nervous, having worry and fear for no reason; 3) feeling stressed during daily routine work and 4) having bad mood for no reason with no interest in anything. Each of these four questions were measured in a five-point scale with corresponding assigned scores (never = 1, seldom = 2, about half the time = 3, usually = 4 and always = 5). Cumulative psychological score for each participant was calculated and categorized into: 4/5–8/9 and more.

Subject were enquired (yes/no) if they were ever diagnosed with any of the following diseases: hypertension, diabetes, hyperlipidemia, cardiovascular diseases (CVD), cerebrovascular diseases (CeVD), cancers, chronic obstructive pulmonary disease (COPD), neck, back and other bone/joint diseases and digestive system diseases. Family history of any of these NCDs (yes/no), was also asked.

### Disease measures

Hypertension of the subjects was diagnosed by measuring two readings (before and after the interview) of diastolic (DBP) and systolic (SBP) blood pressure. Hypertension was defined as SBP ≥ 140 mm of Hg and DBP ≥90 mm of Hg in either readings, or having prior diagnosis of hypertension.

After overnight (12 h) fasting, five ml of venous blood was collected from each participant by a nurse for measuring fasting plasma glucose (using GOD-PAD enzymatic kit, Mike Biotechnology Co., Ltd., Sichuan) and lipids (using Total Cholesterol (TC) Assay Kit and Triglycerides (TG) Assay Kit by GOD-PAD enzymatic kit (Mike Biotechnology Co., Ltd., Sichuan)), after processed. Participants having past diagnosis of diabetes or fasting plasma glucose >6.7 mmol/L were defined as diabetic. Hyperlipidemia was definesd as triglycerides level ≥2.30 mmol/L or total cholesterol ≥ 5.72 mmol/L or having pre-diagnosed hyperlipidemia. Other NCDs were defined based on self-reported prior diagnoses which were all confirmed by rechecking the medical records.

After the interview the disease screening results were revealed to the participants in due course of time. Screened-positives subjects were referred to designated treatment centers for respective NCDs.

### Data analysis

Data was double-entered using the software EpiData 3.0 [12] and multiple logic checks to ensure the data quality. SAS version 9.1 [13] was used for all statistical analyses. Descriptive analyses were conducted to determine the distribution of the demographic factors, behaviors and to calculate the prevalence proportions of different kind of NCDs. Participants were categorized into four groups based on the numbers of NCDs they had: having no NCD, having one NCD, having two NCDs and having three or more NCDs. To assess the strength and direction of the association between NCDs and their potential correlates, simple ordinal logistic regressions were performed for univariate analysis [Odds ratio (OR) and 95 % CI]. Multivariate ordinal logistic regressions were conducted next, adjusting for age (continuous), gender, marital status and education.

### Ethical statement

The study process and content were approved by the Ethics Committee of Shijiazhaung Center for Disease Control and Prevention. Signed informed consent was obtained from each participant prior to the interview and blood collection. If the participants were less than 18 years old, their parents or other guardians signed the informed consent forms. Each of the participants had the discretion to freely decline or withdraw from this survey at any point of time.

## Results

### Demographics and behaviors

In this comprehensive cross-sectional study, an overall 6003 participants from rural areas of Shijiazhuang, Hebei, China were recruited, with a response rate of 97.6 % (6003/6150). The mean age for the 6003 participants was 37.4 ± 14.8. Among these participants, 2836 were male (about 47.2 %) and 3167 were female (52.8 %). About 23 % of the participants (22.7 % among male and 23.3 % among female) were aged more than 50 years, and more than two third (77.6 %) of the participants were married (75.1 % and 79.9 % among male and female respectively). Approximately 65 % of the subjects attended junior/senior or senior high school (Table 1).

**Table 1:** Demographic characteristics, anthropometry and behaviors of participating rural residents (aged 15 years or more) in Shijiazhuang, Heibei, China (N = 6003), 2011

Variables		Male (*n* = 2836)		Female (*n* = 3167)		Total^a^(*N* = 6003)	
		Frequency	Percent (95 % CI)	Frequency	Percent (95 % CI)	Frequency	Percent
Age (years)	Less than 30	1128	39.8 (38.0, 41.6)	1128	35.6 (33.9,37.3)	2256	37.6
	30 to 39	559	19.7 (18.2, 21.2)	652	20.6 (19.2, 22.0)	1211	20.2
	40 to 49	506	17.8 (16.4,19.2)	649	20.5 (19.1, 21.9)	1155	19.2
	50 or more	643	22.7 (21.1, 24.2)	738	23.3 (21.8, 24.8)	1381	23.0
Marital status	Never married	672	23.7 (22.1, 25.3)	579	18.3 (16.9, 19.6)	1251	20.8
	Widowed or Divorced	34	1.2 (0.8, 1.6)	58	1.8 (1.4, 2.3)	92	1.5
	Married	2130	75.1 (73.5, 76.7)	2530	79.9 (78.5, 81.3)	4660	77.6
Education	Elementary school or less	771	27.2 (25.5, 28.8)	1192	37.6 (35.9, 39.3)	1963	32.7
	Junior or senior high school	2003	70.6 (69.0, 72.3)	1909	60.3(58.6, 62.0)	3912	65.2
	College or above	62	2.2 (1.6, 2.7)	66	2.1 (1.6, 2.6)	128	2.1
BMI (kg/m^2^)	18.5 or less	166	5.9 (5.0, 6.7)	120	3.8 (3.1, 4.4)	286	4.8
	18.6-24.9	1633	57.7 (55.8, 59.5)	1708	54.0 (52.2, 55.7)	3341	55.7
	25 or above	1033	36.5 (34.7, 38.2)	1336	42.2 (40.5, 43.9)	2369	39.5
Active smoking status during the past 12 months	Daily smoker	1177	41.5 (39.7, 43.3)	10	0.3 (0.1, 0.5)	1187	19.8
	Smoker but not daily	264	9.3 (8.2, 10.4)	5	0.2 (0.02, 0.3)	269	4.5
	Ex-smoker	151	5.3 (4.5, 6.2)	3	0. 1 (0.0, 0.2)	154	2.6
	Never smoker	1243	43.8 (42.0, 45.7)	3149	99.4 (99.2, 99.7)	4392	73.2
Passive smoking in the past 12 months	No	1650	58.2 (56.4, 60.0)	1856	58.6	3506	58.4
	Yes	1186	41.8 (40.0, 43.6)	1311	41.4	2497	41.6
Alcohol consumption in the past 12 months	Yes, every day	276	9.7 (8.6, 10.8)	4	0.1 (0.0, 0.2)	280	4.7
	Not every day	1289	45.5 (43.6, 47.3)	139	4.4 (3.7, 5.1)	1428	23.8
	Ex-drinker	75	2.6 (2.0, 3.2)	2	0.1 (0.0, 0.2)	77	1.3
	Never drinker	1195	42.2 (40.3, 44.0)	3022	95.4 (94.7, 96.1)	4217	70.3
Breakfast eating in the past 12 months	Every morning	2430	85.7 (84.4, 87.0)	2803	88.5 (87.4, 89.6)	5233	87.2
	5 to 6 times per week	65	2.3 (1.7, 2.8)	80	2.5 (2.0, 3.1)	145	2.4
	3-4 times per week	147	5.2 (4.4, 6.0)	131	4.1 (3.4, 4.8)	278	4.6
	1-2 times per week	66	2.3 (1.8, 2.9)	56	1.8 (1.3, 2.2)	122	2.0
	Never	127	4.5 (3.7, 5.2)	96	3.0 (2.4, 3.6)	223	3.7
Labor work in the farm during the past 12 months	Never	686	24.2 (22.6, 25.8)	1028	32.5 (30.8, 34.1)	1714	28.6
	Yes, but no busy season	569	20.1 (18.6, 21.5)	601	19.0 (17.6, 20.3)	1170	19.5
	Yes, and have busy season	1580	55.7 (53.9, 57.6)	1537	48.6 (46.8, 50.3)	3117	51.9
Exercise in last 12 months	Often	275	9.7 (8.6, 10.8)	272	8.6 (7.6, 9.6)	547	9.1
	Rare	245	8.6 (7.6, 9.7)	243	7.7 (6.7, 8.6)	488	8.1
	Never	2315	81.7 (80.2, 83.1)	2651	83.7 (82.4, 85.0)	4966	82.8
Weight Change in last 12 months	Increased more than 2.5kgs	346	12.2 (11.0, 13.4)	507	16.1 (14.8, 17.4)	853	14.3
	Not changed	2235	79.1 (77.6, 80.6)	2318	73.6 (72.0, 75.1)	4553	76.2
	Decreased more than 2.5kgs	244	8.6 (7.6, 9.7)	326	10.3 (9.3, 11.4)	570	9.5
Psychological Score	4	890	31.4 (29.7, 33.1)	836	26.4 (24.9, 27.9)	1726	28.8
	5-8	1320	46.5 (44.7, 48.4)	1438	45.4 (43.7, 47.1)	2758	45.9
	9 and above	626	22.1 (20.5, 23.6)	893	28.2 (26.6, 29.7)	1519	25.3

Note: ^a^Some variables have the problem of missing data, and the total number is less than 6003

About 40 % of the participating rural residents had BMI ≥25 (36.5 % among male and 42.2 % among female), while in last 12 months: 20 % smoked every day (41.5 % among male and 0.3 % among female), 42 % experienced passive smoking and about 5 % consumed alcohol everyday (9.7 % among male and 0.1 % among female). It was also revealed that in the last 12 months period, about 4 % of the participants never ate breakfast, 83 % never did any exercise and about one quarter of them had psychological score of more than 8 points indicating poor mental health.

### Prevalence of NCDs

Among recruited persons, 3068 participants were diagnosed with hypertension either previously or during the study (based on self-report and measurement), with an overall prevalence of 51.1 % (95 % CI: 50.5-52.4, 51.0 % in male and 51.2 % in female). Previously or during the study, according to the self-report of the participants, 404 (6.7 %, 95 % CI: 6.1-7.4) participants were diagnosed as diabetic and 554 (9.2 %, 95 % CI: 8.5-10.0) participants were diagnosed with hyperlipidemia. Besides these, 272 (4.5 %, 95 % CI: 4.0-5.1) of the participants had CVDs, 139 (2.3 %, 95 % CI: 2.0–2.7) had CeVDs, 13 (0.2 %, 95 % CI: 0.1–0.4) had cancers, 120 (2.0 %, 95 % CI: 1.7–2.4) had COPD, 729 (12.1 %, 95 % CI: 11.3–13.0) had neck, back or other bone and joint diseases and 470 (7.8 %, 95 % CI 7.2–8.5) had digestive system diseases (self- report, Table 2).

**Table 2:** Prevalence of non-communicable diseases (NCDs) of participating rural area residents (15 or older) in Shijiazhuang, Heibei, China (N = 6003), 2011

Variable		Male (*n* = 2836)		Female (*n* = 3167)		Total^a^(*N* = 6003)	
		Frequency	Percent	Frequency	Percent	Frequency	Percent (95 % CI)
High Blood Pressure	Yes	1446	51.0	1622	51.2	3068	51.1 (50.5, 52.4)
	No	1390	49.0	1545	48.8	2935	48.9 (47.6, 49.5)
Diabetes	Yes	173	6.1	231	7.3	404	6.7 (6.1, 7.4)
	No	2663	93.9	2936	92.7	5599	93.3 (92.6, 93.9)
Hyperlipidemia	Yes	261	9.2	293	9.3	554	9.2 (8.5, 10.0)
	No	2574	90.8	2874	90.7	5448	90.8 (90.0, 91.5)
Cardiovascular diseases	Yes	122	4.3	150	4.7	272	4.5 (4.0, 5.1)
	No	2714	95.7	3017	95.3	5731	95.5 (94.9, 96.0)
Cerebrovascular diseases	Yes	55	1.9	84	2.6	139	2.3 (2.0, 2.7)
	No	2781	98.1	3083	97.4	5864	97.7 (97.3, 98.0)
Cancer	Yes	5	0.2	8	0.2	13	0.2 (0.1. 0.4)
	No	2831	99.8	3159	99.8	5990	99.8 (99.6, 99.9)
Chronic obstructive pulmonary disease	Yes	72	2.5	48	1.5	120	2.0 (1.7, 2.4)
	No	2764	97.5	3119	98.5	5883	98.0 (97.6, 98.3)
Neck, back & other bone/joint diseases	Yes	307	10.8	422	13.3	729	12.1 (11.3, 13.0)
	No	2529	89.2	2745	86.7	5274	87.7 (87.0, 88.7)
Digestive system diseases	Yes	234	8.2	236	7.4	470	7.8 (7.2, 8.5)
	No	2602	91.8	2931	92.6	5533	92.2 (91.5, 92.8)
Overall NCDs	No NCD	1054	37.2	1171	37.0	2225	37.1 (35.8, 38.3)
	One NCD	1252	44.2	1328	41.9	2580	43.0 (41.7, 44.2)
	Two NCDs	389	13.7	477	15.1	866	14.4 (13.5, 15.3)
	Three or more NCDs	140	4.9	191	6.0	331	5.5 (5.0, 6.1)

Note: ^a^Some variables have the problem of missing data, and the total number is less than 6003

Overall, 43.0 % (95 % CI: 41.7–44.2) of the participants had one kind of NCDs (44.2 % among male and 41.93 % among female), 14.4 % (95 % CI: 13.5–15.3) had two kinds of NCDs, and 5.5 % (95 % CI: 5.0–6.1) had three or more kinds of NCDs.

**Fig. 1:**
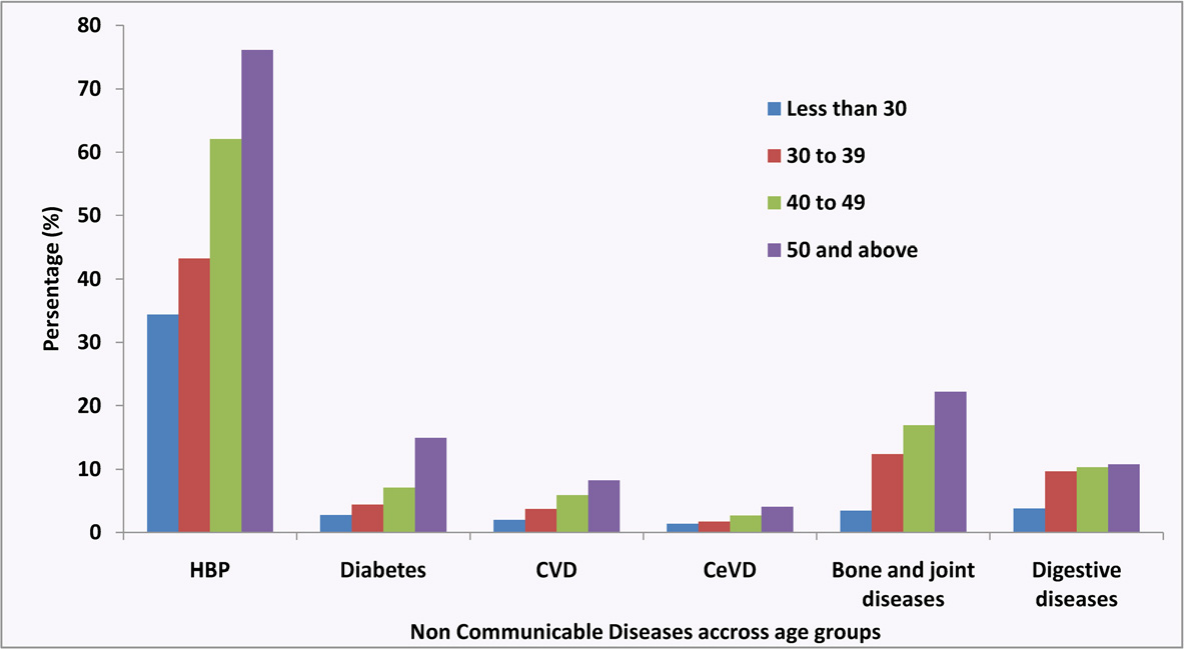
Prevalence of different kind of NCDs in different age groups among the participants from rural area of Shijiazhuang, Hebei, China (N = 6003), 2011

Figure 1 further pointed out that with increasing age, all the proportions of participants with hypertension, diabetes, CVD, CeVD, bone and joint diseases, and digestive diseases did increase.

### Correlates of NCDs

Both crude and adjusted models indicated that compared to the participants with BMI between 18.6 to 24.9, recruited participants with BMI ≥25 were more likely to suffer from increased number of NCDs (Crude OR = 2.31, 95 % CI: 2.09–2.56 while adjusted Odds Ratio: AOR = 1.60, 95 % CI: 1.44–1.78). Crude model did also show that the participants with BMI of 18.5 or less, seemed to have the lowest odds of NCDs acquisition (Crude OR = 0.68, 95 % CI: 0.54–0.86) (Table 3).

**Table 3:** Strength of association between Non-communicable-diseases (NCDs) and their correlates, from ordinal logistic regression, among rural residents of Shijiazhuang, Hebei, China (n = 6003), 2011

Variable		Crude model		Adjusted Model^a^	
		Crude OR	95 % CI	AOR	95 % CI
BMI	18.5 or less	0.68	0.54, 0.86	1.17	0.91, 1.50
	18.6 to 24.9	Reference		Reference	
	25 and above	2.31	2.09, 2.56	1.60	1.44, 1.78
	Continuous	1.10	1.09,1.12	1.04	1.03,1.05
Family History	No	Reference		Reference	
	Yes	1.18	1.07, 1.30	1.08	0.98, 1.20
Active Smoking in last 12 months	Daily smoker	1.18	1.05, 1.33	1.06	0.90, 1.23
	Smoker but not daily	1.00	0.80, 1.26	1.16	0.90, 1.49
	Ex-smoker	2.64	1.96, 3.54	1.54	1.12, 2.11
	Never smoker	Reference		Reference	
Passive Smoking in last 12 months	No	Reference		Reference	
	Yes	1.29	1.18, 1.42	1.33	1.20, 1.47
Exercise in last 12 months	Often	Reference		Reference	
	Rare	1.25	1.00, 1.58	1.29	1.02, 1.64
	Never	1.70	1.44, 2.01	1.06	0.87, 1.28
Weight Change in last 12 months	Increased more than 2.5 kgs	1.11	0.97, 1.28	1.39	1.21, 1.60
	Not changed	Reference		Reference	
	Decreased more than 2.5 kgs	0.96	0.82, 1.12	1.30	1.10, 1.54
Alcohol consumption in last 12 months	Yes, every day	1.43	1.14, 1.79	1.15	0.90, 1.48
	Not every day	0.99	0.89, 1.11	1.16	1.01, 1.33
	Ex-drinker	3.21	2.12, 4.84	1.94	1.27, 2.98
	Never drinker	Reference		Reference	
Breakfast Eating in last 12 months	Every morning	Reference		Reference	
	5-6 times per week	0.61	0.44, 0.83	1.01	0.73, 1.40
	3-4 times per week	0.83	0.66, 1.04	1.37	1.08, 1.72
	1-2 times per week	0.74	0.52, 1.03	1.13	0.80, 1.60
	Never	1.18	0.92, 1.51	1.88	1.46, 2.43
Labor Work in the farm during last 12 months	Never	Reference		Reference	
	Yes, but no busy season	1.00	0.87, 1.15	0.90	0.78, 1.04
	Yes, and have busy season	1.36	1.22, 1.52	1.10	0.98, 1.24
Psychological Scores	4	Reference		Reference	
	5-8	1.10	0.98, 1.24	1.14	1.01, 1.44
	9 and above	1.36	1.20, 1.55	1.26	1.10, 1.28

Note:^a^model adjusted for age, gender, education and marital status

*COR* Crude Odds Ratio, *AOR* Adjusted Odds Ratio, *CI* Confidence Intervals

Compared to those who had no family history of NCDs, participants with family history (Crude OR = 1.18, 95 % CI: 1.07–1.30; AOR = 1.08, 95 % CI: 0.98–1.20) had higher odds of having more NCDs, although the adjusted result are marginal significant. With reference to those who never smoked, daily smokers (Crude OR = 1.18, 95 % CI: 1.05–1.33; AOR = 1.06, 95 % CI: 0.90–1.23) and ex-smokers (Crude OR = 2.64, 95 % CI: 1.96–3.54; AOR = 1.54, 95 % CI: 1.12–2.11) had higher odds of suffering from more NCDs. The crude and adjusted model also demonstrated that passive smoking was significantly associated with higher odds of having more NCDs (Crude OR = 1.29, 95 % CI: 1.18–1.42; AOR = 1.33, 95 % CI: 1.20–1.47). Similar to smoking, compared to those who never drank alcohol, daily drinkers (Crude OR = 1.43, 95 % CI: 1.14–1.79; AOR = 1.15, 95 % CI: 0.90–1.48) and ex-drinkers (Crude OR = 3.21, 95 % CI: 2.12–4.84; AOR = 1.94, 95 % CI: 1.27–2.98) had higher odds of suffering from more NCDs.

In comparison with those who did exercise every day, participants exercising rarely (Crude OR = 1.25, 95 % CI: 1.00–1.58; AOR = 1.29, 95 % CI: 1.02–1.64) and those who never did any exercise (Crude OR = 1.70, 95%CI: 1.44–2.01; AOR = 1.06, 95 % CI: 0.87–1.28) seemed to have higher risk of developing more NCDs, although the results for adjusted model is not significant for the later.

Adjusted model pointed out that having significant weight change in the past 12 months was associated with increased likelihood for having. After adjustment, compared to those who ate breakfast every day, participants who never ate breakfast in the past 12 months had significantly higher odds of suffering from more NCDs, (AOR = 1.88 (95 % CI: 1.46–2.43). Additionally, poorer mental health as indicated by higher psychological scores, also increased the risk of having more NCDs.

## Discussion

In this study, we found that the prevalence of hypertension increased significantly with increasing age, corroborating with prior findings [14]. The observed overall proportion of hypertensives in our study was higher than the findings from one study conducted among rural adults (44.1 % (48.7 % for male and 39.6 % for female)) in Liaoning province between 2004 and 2006 [15]. This observed prevalence was also much higher than the age-standardized national prevalence of hypertension among Chinese (17.7 %) reported in 2002 [16]. High prevalence of hypertension among rural residents aged 15 or more in Shijiazhuang thus revealed that residents of this study area were facing a worsening epidemic of hypertension. Previous studies already demonstrated that hypertension is often associated with increased risks of stroke, ischemic heart diseases, hypertensive complications and other cardiovascular disease [17, 18]. The diabetes prevalence was observed to be lower than the age-standardized prevalence of diabetes reported by a national survey among Chinese adults [19], even the sampling strategy and study population may different. This difference might be explained by the variation in the burden of diabetes across age groups and geographic regions (difference between urban and rural areas) in China [20]. Despite being a bit lower than the national estimate, diabetes prevalence as observed in our study still indicated towards a huge health and economic burden, additional to the increased likelihood of developing complications of diabetes and resultant risk of premature death [19].

Besides hypertension and diabetes, our study also found significantly higher prevalence of hyperlipidemia among rural residents in the selected rural population. Being an established risk factor for CVD, this increased burden of hyperlipidemia might explain the observation that 4.5 % (8.3 % among participants who were aged 50 years or more) of the study subjects had pre-diagnosed CVD [17].

In our study, we also found the participants had higher CeVD, cancers, COPD and other NCDs. More importantly, more than 60 % of the participants had at least one kind of NCDs while about 20 % had two or more NCDs which indicated that burden of NCDs in this population had reached epidemic proportions. Keeping in mind the fact that NCDs account for 60 % of all deaths globally, [21], this huge burden of NCDs in this representative rural population of China points towards a worrisome public health challenge in this country.

The results of our study demonstrated that higher BMI was significantly correlated with increased likelihood of having NCDs. Similar findings were reported by several prior studies revealing the role of increased BMI in increasing the risk of hypertension, diabetes, CVD and other NCDs [22–24], while there could be differences in methodology and population characteristics between our study and others. These results probably indicated towards the need for an effective intervention strategy to increase awareness and improve practice in terms of healthy dietary modifications among rural residents of china, to prevent and control these epidemics of NCDs.

Alike others [25], our results also indicated that active smoking, passive smoking and alcohol drinking were strong correlates for the development of NCDs. These findings probably emphasized the need for more in-depth studies among rural residents of China, to identify which factor is a significant contributor.

In this study population, ex-smokers and ex-drinkers were found to have higher likelihood of having more NCDs. Reverse causation could be considered as one of the potential explanations for this phenomenon, as participants who already diagnosed with any NCD were very likely to be suggested by the doctors to quit smoking and alcohol drinking.

Lack of physical activity was associated with increased risk of having NCDs, corroborating with the findings of several studies conducted before [25, 26]. Since it has already been established that physical inactivity increases the risk of NCDs, and shortens life expectancy, motivational interventions to improve healthy lifestyles seem to be required urgently in this population to promote regular physical exercise [26]. Alike another study conducted in New Zealand [27], we observed that skipping breakfast was positively correlated with higher risk of developing NCDs.

The adjusted model did show that participants who experienced significant weight change in the past 12 months (either increased or decreased) had higher risk of developing NCDs. Reverse causation could well be one reason for this finding also, as NCDs might have lead to the weight change of the participants. However, due to the cross-sectional design of our study, we can not comment further on this observation regarding the temporal direction of this association. Our study also indicated that poorer mental health (depicted by higher psychological score) was significantly correlated with higher risk of having NCDs.

According to our knowledge this was the first comprehensive study in rural Shijiazhuang to determine the association of NCDs with their potential predictors. By virtue of its sampling design this study was able to recruit a representative population of rural residents (aged 15 years or more). The measured prevalence of NCDs as well as the observed associations of NCDs with their potential predictor can thus be extrapolated by the policy-makers for the purpose of designing appropriate targeted interventions. Large sample size, use of biological markers, advanced laboratory investigation techniques and following uniform study protocol by virtue of extensive training of all study personnel to minimize interviewer bias were the major strengths of this study. Moreover, the use of more efficient statistical analyses in the form of ordinal logistic regression can also be considered as an additional strength of this study.

As an observational study, our study had several limitations. Because of the cross-sectional design, temporal ambiguity prevented us from drawing causal inferences based on our results and we recommend that any such interpretation should be made with caution. Vulnerability of the self-reported information to social desirability bias, might lead to misclassification in our study. Specifically, due to budgetary constraints, we had to depend on self-reporting for the diagnosis of some outcomes (CVD, CeVD etc.) which might have lead to the misclassification of outcomes, and introduced information bias in our study. Besides, the number of NCDs for each participant were based on both self-report and measurements, while the sensitivity for these two methods may different, which may further lead to the problem of misclassification. Although we expect these misclassifications to be minimal as we reconfirmed the self-reported diagnoses with available medical records in most of the occasions. Selection bias and lack of generalizability were other likely shortcomings. As it was not possible for us to have an exhaustive questionnaire, information was collected only on selected behaviors and covariates leading to the possibilities of residual confounding. We also could not establish the validity of the method of measuring the mental health status in our study, thus, we did not includemental health problems as NCDs. Last but not least, this study was conducted few years ago (2010–2011), thus requirement for fresh data is probably there.

## Conclusion

Even with these limitations, we can still conclude that the prevalence of NCDs, especially of hypertension, diabetes and hyperlipidmia were high among rural residents aged 15 years or more in Shijiazhuang, Hebei, China. These NCDs were highly correlated with unhealthy behaviors and life styles like smoking, alcohol drinking, lack of physical exercise, skipping breakfasts etc. Implementation of targeted intervention strategies like raising tobacco and alcohol taxes, legislation of health warnings, enactment of laws to maintain smoke-free work environment, undertake motivational programs to induce healthy life styles and improve awareness seemed to be urgently required in this population to change the behaviors of the residents and thus to minimize the risk of NCDs.
